# Characterization of the complete plastome of *Elymus tangutorum* (Poaceae: Triticeae)

**DOI:** 10.1080/23802359.2019.1674217

**Published:** 2019-10-04

**Authors:** Meiling Jing, Yushou Ma, Huimei Li, Jiuli Wang

**Affiliations:** a Key Laboratory of Biotechnology and Analysis and Test in Qinghai-Tibet Plateau, College of Ecological Environment and Resources, Qinghai Nationalities University, Xining, China;; b State Key Laboratory of Plateau and Agriculture, Qinghai Academy of Animal and Veterinary Science, Qinghai University, Xingning, China

**Keywords:** *Elymus tangutorum*, plastome, Triticeae, Poaceae

## Abstract

*Elymus tangutorum* (Nevski) Handel-Mazzetti (Poaceae: Triticeae), a hexaploid perennial herb, is a kind of forage plant with large biomass. In this study, the complete plastome sequence of *E. tangutorum* was reported. The size of the plastome is 134,949 bp in length, including a large single copy region (LSC) of 80,556 bp, a small single copy region (SSC) of 12,767 bp, and a pair of inverted repeat (IR) regions with 20,813 bp. Moreover, a total of 131 functional genes were annotated, including 85 protein-coding genes, 38 tRNA genes, and 8 rRNA genes. The maximum likelihood (ML) phylogenetic tree suggested that *E. tangutorum* was closely related to *Elymus libanoticus* and *Dasypyrum villosum*.

*Elymus tangutorum* (Nevski) Handel-Mazzetti (Poaceae: Triticeae) is a hexaploid perennial herb distributing in steppes and mountain slopes in China, Bhutan, and Nepal. It provides good forage for cattle and sheep in northern China (Chen and Zhu 2006). The plastome is valuable in plant systematics research due to its highly conserved structures, uniparental inheritance, and haploid nature (Fu et al. 2016). Plastome have also been smartly engineered to confer useful agronomic traits and/or serve as bioreactors (Jin and Daniell 2015). Here, the complete plastome of *E. tangutorum* (Genbank accession number: MN420499) was sequenced, which will provide genomic and genetic sources for further research.

The fresh, young leaves of *E. tangutorum* were collected from Guoluo state, Qinghai Province, China (100°13′6.5″E, 34°27′56.9″N). Its total genomic DNA was extracted from the fresh leaves (about 1.5 g) with a modified CTAB method (Doyle and Doyle 1987). The voucher specimen was kept in Herbarium of the Northwest Institute of Plateau Biology (HNWP, Jing20190518), Chinese Academy of Sciences. The experiment and analysis scheme refers to Wang et al. (2019). Genome sequencing was performed using the Illumina HiSeq Platform (Illumina, San Diego, CA) at Genepioneer Biotechnologies Inc., Nanjing, China. Approximately 7.62 GB of clean data were yielded. The trimmed reads were mainly assembled by SPAdes (Bankevich et al. 2012). The assembled genome was annotated using CpGAVAS (Liu et al. 2012).

The size of the plastome is 134,949 bp in length, including a large single copy region (LSC) of 80,556 bp, a small single copy region (SSC) of 12,767 bp, and a pair of inverted repeat (IR) regions with 20,813 bp. Moreover, a total of 131 functional genes were annotated, including 85 protein-coding genes, 38 tRNA genes, and 8 rRNA genes. The protein-coding genes, tRNA genes, and rRNA genes account for 64.89%, 29.00%, and 6.11% of all annotated genes, respectively.

The maximum likelihood phylogenetic tree was generated based on the plastome of *E. tangutorum* and other 19 species of the family Poaceae (Figure 1). Alignment was conducted using MAFFT (Katoh and Standley 2013). The phylogenetic tree was built using RAxML (Stamatakis 2014) with bootstrap set to 1000. The results showed that *E. tangutorum* was closely related to *Elymus libanoticus* and *Dasypyrum villosum*. This study could lay a foundation for chloroplast genome engineering of *E. tangutorum* and its allies in the future.

**Figure 1.**
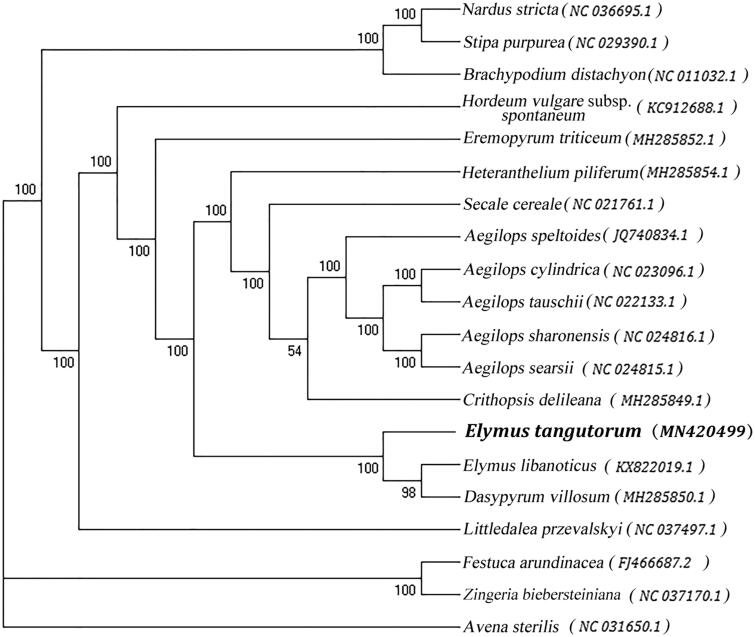
The maximum likelihood phylogenetic tree based on 20 chloroplast genomes from the family Poaceae.
